# Anthropogenic transformations of river ecosystems are not always bad for the environment: Multi-taxa analyses of changes in aquatic and terrestrial environments after dredging of a small lowland river

**DOI:** 10.7717/peerj.12224

**Published:** 2021-09-29

**Authors:** Robert Stryjecki, Andrzej Zawal, Tomasz Krepski, Edyta Stępień, Edyta Buczyńska, Paweł Buczyński, Stanisław Czachorowski, Łukasz Jankowiak, Joanna Pakulnicka, Anna Sulikowska-Drozd, Vladimir Pešić, Grzegorz Michoński, Michał Grabowski, Aleksandra Jabłońska, Magdalena Achrem, Tomasz Olechwir, Lech Pietrzak, Agnieszka Szlauer-Łukaszewska

**Affiliations:** 1 Department of Zoology and Animal Ecology, University of Life Sciences in Lublin, Lublin, Poland; 2 Institute of Marine and Environmental Science, University of Szczecin, Szczecin, Poland; 3 Institute of Biology, University of Szczecin, Szczecin, Poland; 4 Department of Zoology and Nature Protection, Maria Curie-Skłodowska University, Lublin, Poland; 5 Department of Ecology and Environmental Protection, University of Warmia and Mazury in Olsztyn, Olsztyn, Poland; 6 Department of Invertebrate Zoology and Hydrobiology, University of Lodz, Łódź, Poland; 7 Department of Biology, University of Montenegro, Podgorica, Montenegro; 8 B.P.P. “Bagnik” Lech Pietrzak, Olsztyn, Poland

**Keywords:** Multi-stage anthropogenic transformation, Habitat diversity, Species diversity, Remedial measures, Invertebrates, Bioindicators

## Abstract

Rivers are one of the most commonly transformed aquatic ecosystems. Most papers present significantly negative effects of activities such as dredging or channel regulation on the ecological status of rivers. The purpose of this work was to compare the response of various groups of invertebrates (Mollusca, Hydrachnidia, Odonata, Heteroptera, Coleoptera and Trichoptera) to an intervention involving dredging in conjunction with the removal of riparian vegetation. Habitat diversity increased after the dredging, and more individuals and species were caught than before the dredging. The increase in habitat diversity after the dredging translated into an increase in the species diversity of most investigated groups. Individual groups of invertebrates showed varied responses to the dredging, depending on the role of the terrestrial phase in their life cycle: the greater the role of the terrestrial phase in the life cycle, the more the group was affected by changes in the terrestrial environment following the intervention. In consequence, the intervention had the greatest negative impact on insects, and among these, on adult Odonata. The following conclusions can be drawn: (1) Dredging can benefit a previously anthropogenically transformed river ecosystem by increasing habitat diversity; (2) Odonata are particularly useful for assessing the impact of this type of intervention on invertebrate communities. They can be considered good indicators of habitat disturbances in both aquatic and terrestrial ecosystems.

## Introduction

Rivers are often anthropogenically transformed, due to various strategies for local management of surface waters, as well as land use. Human-induced changes in river ecosystems usually have many negative effects for both the river biotope and its biocenosis. Bączyk et al. (2018) analyzed 203 publications on anthropogenic transformation of lowland rivers and found that the vast majority of the studies showed negative effects of human interference in the river ecosystem, resulting in deterioration of the ecological status of rivers and significant changes in the qualitative and quantitative composition of plant and animal communities. Very few studies revealed no negative effects on river ecosystems or reported positive changes (see Bączyk et al. (2018) for the review).

One of the human activities leading to transformations of river ecosystems is dredging. Dredging has a substantial impact on the river ecosystem, as it entails changes in numerous environmental features (such as water flow velocity, bottom sediment structure, the degree of bottom coverage by aquatic vegetation, and spatial habitat structure), which in turn generate changes in the invertebrate fauna (Wilber & Clark, 2007; Kändler & Seidler, 2013; Benitez-Mora & Camargo, 2014). Interventions such as removing macrophytes and fallen tree branches and trunks from the river bed, removing layers of sediments and altering their structure, and modifying river channel features are likely to have long-lasting effects for the functioning of the ecosystem. These modifications and transformations alter river ecosystems by disrupting the river continuum, reducing the number of mesohabitats, decreasing habitat diversity, and changing the spatial arrangement of habitats (Rader, Voelz & Ward, 2008; Crook et al., 2015).

The responses of invertebrate fauna to human-induced disturbances in river ecosystems are complex and can vary depending on the habitat structure and level of stress (Fjellheim et al., 1993). Regeneration of riverine biocenoses following human-made disturbances can take place at different rates and in different directions. Responses to maintenance interventions may include a decline in the abundance of some stenotopic species, an increase in that of eurytopic species, the appearance of new species, and the disappearance of some that were present before the transformation (see Bączyk et al. (2018) for a review). Such responses were described in a series of papers dealing with specific groups of aquatic invertebrates (Mollusca, Hydrachnidia, Odonata, Heteroptera, Coleoptera and Trichoptera) in the River Krąpiel, which was subjected to dredging (Zawal et al., 2015; Buczyński et al., 2016; Dąbkowski et al., 2016; Płaska et al., 2016; Zawal et al., 2016a, 2016b).

As typical dredging procedures are carried out in the aquatic environment, most studies concerning this type of intervention focus on changes in environmental conditions in the aquatic environment and on the impact of these changes on aquatic invertebrates (Kaenel & Uehlinger, 1999; de Jonge & de Jong, 2002; Wilber & Clark, 2007; Kändler & Seidler, 2013). In some cases, however, dredging is accompanied by related activity in the terrestrial environment, such as clearing of trees and bushes occupying the banks of the river (Stępień et al., 2015). Some groups of invertebrates inhabiting river ecosystems (mainly winged stream insects, but also others, such as water mites) explore the terrestrial environment at certain stages of their lives (Corbet, 1999; Davids et al., 2007). Most aquatic insects must leave the water to complete their life cycle, and then they become an integral part of the terrestrial biocenosis (Jackson & Resh, 1989). The presence of adult aquatic insects in the terrestrial areas near streams maintains the connection between aquatic and terrestrial environments and creates a variety of dependencies between these two environments (Malmqvist, 2002; Raitif, Plantegenest & Roussel, 2019). Due to the connection between aquatic and terrestrial environments, in the case of animals whose life cycle includes aquatic and aerial stages, it is large-scale processes (such as long-distance migration of adults) rather than local ones that explain the population dynamics of stream and river insect communities (Peckarsky, Taylor & Caudill, 2000). Changes in the terrestrial environment, such as a reduction in riparian vegetation, can affect invertebrates inhabiting the aquatic environment (Quinn et al., 1992). Therefore, analysis of changes in invertebrate communities inhabiting river ecosystems should also take into account the environmental conditions in the terrestrial environment.

In the case of the River Krąpiel, which is the subject of the present study, in addition to the dredging itself, *i.e.*, the removal of all vegetation from the river bed, the rushes and shrubs on both sides of the river were removed as well (Zawal et al., 2015). As a result of this comprehensive intervention, habitats were altered in both the aquatic environment, *i.e.*, the river channel, and the terrestrial environment, *i.e.*, the river banks (see “Materials & Methods” for more detail). In previously published papers dealing with various groups of aquatic invertebrates in the River Krąpiel, the analyses, discussion and conclusions regarding the effect of dredging on invertebrate fauna were limited to the aquatic environment (Zawal et al., 2015; Buczyński et al., 2016; Dąbkowski et al., 2016; Płaska et al., 2016; Zawal et al., 2016a, 2016b). Those separate papers each focused on a single group of invertebrates, without comparing one group with another, which could have revealed the combined response of the entire biocenosis to dredging. Although the intervention was carried out in both the aquatic environment and the terrestrial environment, the previously published papers contain no references to changes in the terrestrial environment that could have affected the analyzed groups of invertebrates. Also, there was no discussion of the occurrence of a terrestrial phase in most of these groups and its potential influence on the reaction of a given group to the intervention.

In this paper we present a comparative study of several groups of invertebrates. Multi-taxa analyses were performed in order to determine which of these groups best reflect changes in the aquatic and terrestrial environment resulting from the intervention carried out in the River Krąpiel. We believe that multi-taxa analyses are of particular importance, as they provide a broader view of the response of biocenoses to the transformation of river ecosystems. Multi-taxa and comparative analyses can be used to identify groups of invertebrates that are more useful than others for assessing the degree of anthropogenic transformations of rivers. Taking into consideration two environments, aquatic and terrestrial, and emphasizing the importance of the changes in the terrestrial environment following the intervention for aquatic invertebrates is a new concept that was not introduced in previous papers on the River Krąpiel. We believe that an analysis of all groups of invertebrates together and the two environments together can more fully elucidate this complex issue–the reaction of the river biocenosis to an intervention carried out in aquatic and terrestrial environments.

The invertebrates used in the present study, in addition to typical hydrobionts (Mollusca, adult Hydrachnidia, the larvae of Odonata, Heteroptera, Coleoptera and Trichoptera), included groups (or developmental stages) exploring both the aquatic and the terrestrial environment (Hydrachnidia larvae and adult Heteroptera and Coleoptera), as well as terrestrial organisms (adult Odonata). We postulated that due to the occurrence of a terrestrial phase in most of the analyzed groups, changes in macroinvertebrate communities after the intervention were affected by transformations in both the aquatic and terrestrial environments. In addition, it was assumed that the role of the terrestrial phase in the life cycle of individual groups of analyzed invertebrates affects the scale of transformation of their communities after dredging.

In this work two research hypotheses were put forward: (1) the response of individual groups of invertebrates to this type of intervention depends on the changes that have taken place in both the aquatic and terrestrial environment; (2) the greater the role of the terrestrial phase in the life cycle of a given group of invertebrates, the more they are affected by changes in the terrestrial environment following the intervention.

The stretch of the River Krąpiel which is the subject of the present study had previously been transformed by human activity. That transformation, carried out about 100 years ago, involved straightening of the river channel and reinforcement of the river banks. As a consequence, the bottom became covered with silt and overgrown with vegetation, habitats associated with fast water current disappeared, and the fauna of most groups of invertebrates was no longer characteristic of rivers (see Zawal et al., 2015; Buczyński et al., 2016; Dąbkowski et al., 2016; Płaska et al., 2016; Zawal et al., 2016a, 2016b). Therefore the aim of the study was to investigate how dredging may affect the invertebrate community of a river previously transformed by human activity, rather than a natural one.

## Materials & methods

### Characteristics of the studied stretch of the Kra˛piel river and a description of the intervention

**Figure 1:**
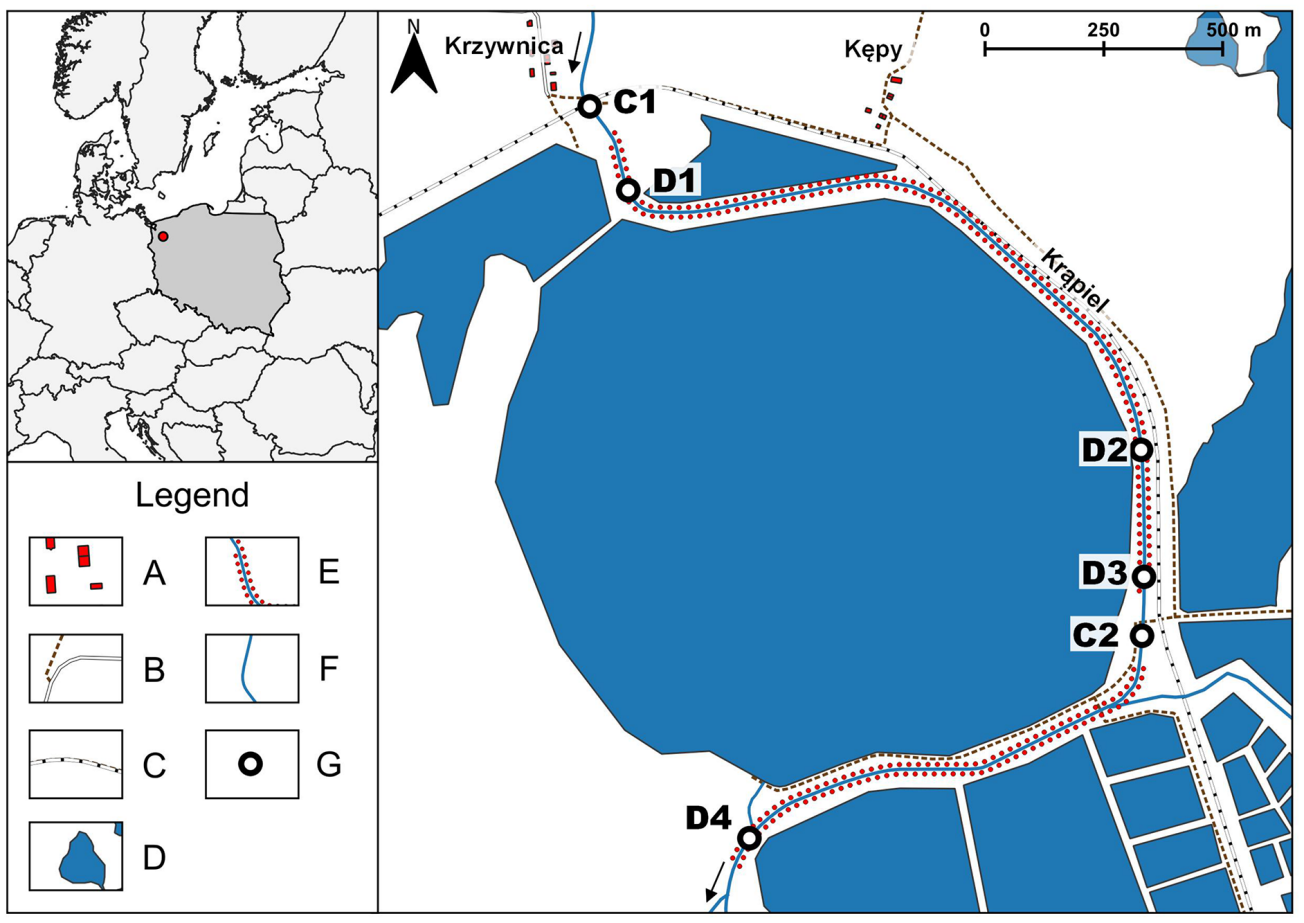
Study area and sampling sites. A–Buildings, B–Roads, C–Railway, D–Fish ponds, E–Dredged sections of the river, F–Non-dredged sections of the river, G–Sampling sites. C1, C2–control (non-dredged) sites, D1–D4–dredged sites.

The River Krąpiel is a small lowland river situated in north-western Poland (Fig. 1). Most of the course of the river is natural, but the research was carried out on a stretch that was previously transformed about 100 years ago. That stretch currently takes the form of a regulated, slow-flowing canal, running alongside fish ponds (Fig. 1). A more detailed description of the morphology of the river and its habitats before the intervention can be found in Stępień et al. (2015), Zawal et al. (2015, 2016a, 2016b), Buczyński et al. (2016), Dąbkowski et al. (2016) and Płaska et al. (2016).

**Figure 2:**
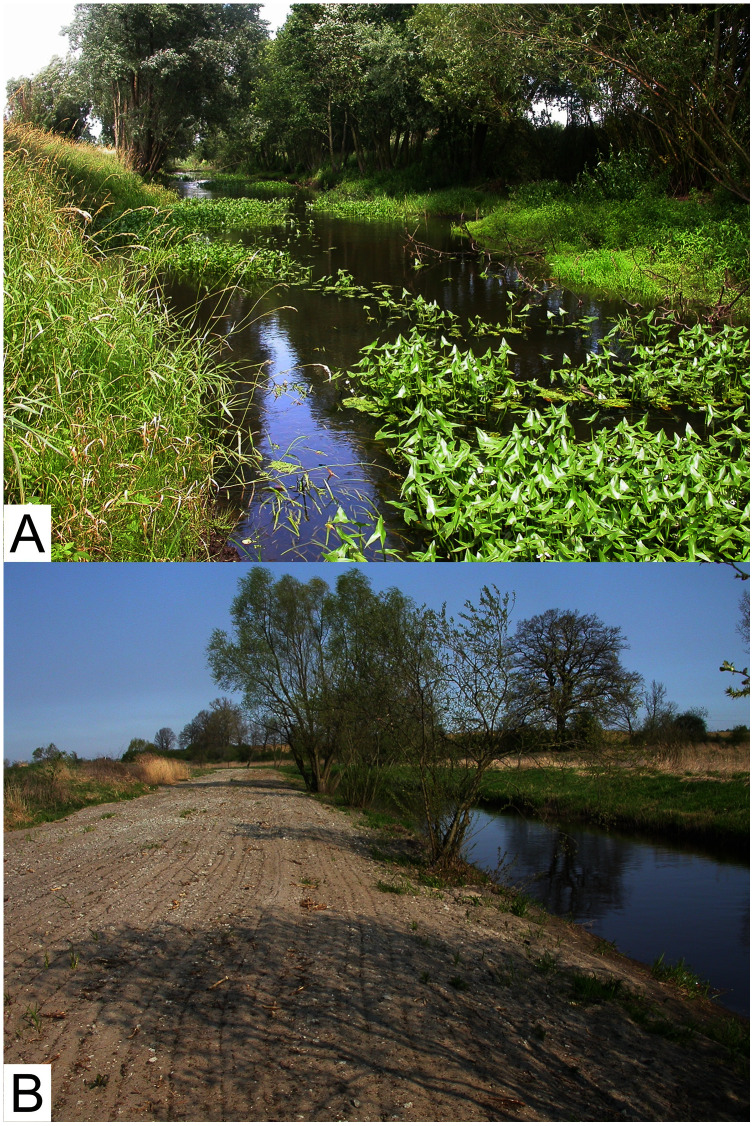
The River Krąpiel before (A) and after (B) the intervention.

The intervention was carried out at the end of January and the beginning of February in 2009. Dredging involved the removal of aquatic vegetation and accumulated silt sediments (Zawal et al., 2015). Following the removal of vegetation, the patency of the channel increased by 20–50% in places that were previously not overgrown and 80% in spots that had been overgrown with reeds (Stępień et al., 2015). Apart from treatments carried out directly in the river bed, a strip of terrestrial vegetation was removed on both sides of the river, leaving only single trees (Fig. 2). About 80–90% of the riparian vegetation was removed at each dredged sampling site. For more details concerning the changes in the aquatic and terrestrial environments of the stretch of the river which is the subject of the present study, see Stępień et al. (2015), Zawal et al. (2015, 2016a, 2016b), Buczyński et al. (2016), Dąbkowski et al. (2016) and Płaska et al. (2016).

### Field procedures

Invertebrate fauna was collected in July 2008 – before dredging, and from April to August 2009 – after dredging. For reasons associated with the plan of action of the company carrying out the project, pre-dredging research was limited to a single sampling. Before-after-control-impact (BACI calculation), comparing 1 month of pre-dredging sampling (July 2008) with only 1 month of post-dredging sampling (July 2009), was used to identify changes in the invertebrate community before and after the dredging, so the number of samples compared was the same. The extended sampling period after the dredging served to observe the rate of recolonization. In this study we did not compare pre-dredging and post-dredging growing seasons, but tracked the recolonization month by month.

On the examined stretch of the river four sites were selected that had been dredged (D1–D4) and two control sites (C1 and C2), where no dredging had been carried out. Dredged and control sites were interspersed within the managed section of the river (Fig. 1). At each site, both dredged and control, samples were taken from two habitats–in the lotic environment (D1/1–D4/1, C1/1 and C2/1) and in the marginal zone (D1/2–D4/2, C1/2 and C2/2).

Aquatic invertebrates were collected using a dip net. Adult Odonata were collected with an entomological net. The methods of hydrobiological and entomological sampling and of processing of the material are described in detail in separate papers on specific systematic groups: Zawal et al. (2015) for Hydrachnidia, Buczyński et al. (2016) for Odonata, Dąbkowski et al. (2016) for Coleoptera, Płaska et al. (2016) for Heteroptera, Zawal et al. (2016a) for Trichoptera, and Zawal et al. (2016b) for Mollusca.

The procedure for assessing biotic and abiotic environmental parameters (water current, the nature of the sediments, bottom coverage by aquatic vegetation) before and after dredging was described in Zawal et al. (2015).

### Calculations

The calculations for the total multi-taxa material were made using the same methods as in previously published papers dealing with individual systematic groups of aquatic invertebrates in the River Krąpiel (Zawal et al., 2015; Buczyński et al., 2016; Dąbkowski et al., 2016; Płaska et al., 2016; Zawal et al., 2016a, 2016b). Before-after-control-impact (BACI) was used to compare the state of biocenoses before and after the dredging (Smith, Orvos & Cairns, 1993). Two parameters were used in the BACI test: abundance and the Shannon–Wiener Index. The statistical analyses used in the study for the total multi-taxa material were as described for Hydrachnidia in Zawal et al. (2015). Statistical analyses were performed using Statistica 9.0 PL software.

## Results

### Changes in habitats

As a result of the dredging, the current velocity and depth increased slightly in the current environments, while the degree of bottom coverage by aquatic plants decreased (Table 1). At some sites, the nature of the sediments changed from organic to mineral. At other sites, patches of mineral sediment appeared among the organic sediments. The removal of vegetation from the banks resulted in a change in sunlight exposure, with partially shaded sites becoming unshaded habitats. The habitat changes were smaller in the environments in the marginal zone than in current environments. There was no change in habitat conditions at the control sites before and after the intervention (Table 1).

**Table 1:** Environmental parameters in habitats of sites along the River Krąpiel.

Site/habitat	Flow (m/s)		Depth (m)		Bottom		Plants (%)		Shadow	
	A	B	A	B	A	B	A	B	A	B
C1/1	0.5	0.46–0.51	0.7	0.7	gravel, stones	gravel, stones	0	0	lack	lack
C1/2	0.01	0.002–0.02	0.5	0.5	sand, silt, mud	sand, silt, mud	70	50–70	partly	partly
D1/1	0.013	0.09–0.16	0.4	0.5	mud	sand, gravel	30	0–10	lack	lack
D1/2	0.01	0.002–0.01	0.2	0.2	silt, mud	sand, silt, mud	90	0–40	partly	lack
D2/1	0.02	0.01–0.05	0.2	0.5	silt, mud	silt, mud	90	0–10	partly	lack
D2/2	0.002	0.001–0.002	0.1	0.2	mud	mud	100	0–40	partly	lack
D3/1	0.02	0.02–0.05	0.3	0.5	silt, mud	sand, silt, mud	20	0–10	partly	lack
D3/2	0.002	0.001–0.002	0.1	0.2	mud	sand, silt, mud	80	0–40	partly	partly
C2/1	0.14	0.09–0.2	0.5	0.5	sand, gravel	sand, gravel	0	0	partly	partly
C2/2	0.003	0.001–0.003	0.2	0.2	sand, mud	sand, mud	70	30–70	partly	partly
D4/1	0.001	0.001–0.003	0.5	0.7	mud	mud	40	0–40	partly	lack
D4/2	0.04	0.03–0.06	0.5	1.0	mud	mud	30	0–20	lack	lack

**Note:**

D1–D4–dredged sites, C1–C2–control (non-dredged) sites, D1/1–D4/1, C1/1–C2/1–lotic environment, D1/2–D4/2, C1/2–C2/2–marginal zone, A–before dredging, B–after dredging.

### Changes in invertebrate assemblages

In the entire study period, a total of 6,591 invertebrate individuals belonging to 230 species were collected (Table 2). Hydrachnidia were the most abundant and species-rich taxon. Other systematic groups were far less numerous and represented by fewer species (Table 2).

**Table 2:** Number of species (n.s.) and number of individuals (n.i.) found in the investigated section of the River Krąpiel.

Taxa	Total		Before dredging		After dredging	
	n.s.	n.i.	n.s.	n.i.	n.s.	n.i.
Mollusca	32	717	18	190	31	527
Hydrachnidia	69	3,895	28	537	63	3,358
Odonata larvae	10	73	7	33	10	40
Odonata adults	23	412	18	282	17	130
Odonata total	25	485	19	315	20	170
Heteroptera	21	545	18	149	18	396
Coleoptera	57	483	28	97	43	386
Trichoptera	26	466	13	162	24	304
TOTAL	230	6,591	124	1,450	199	5,141

**Figure 3:**
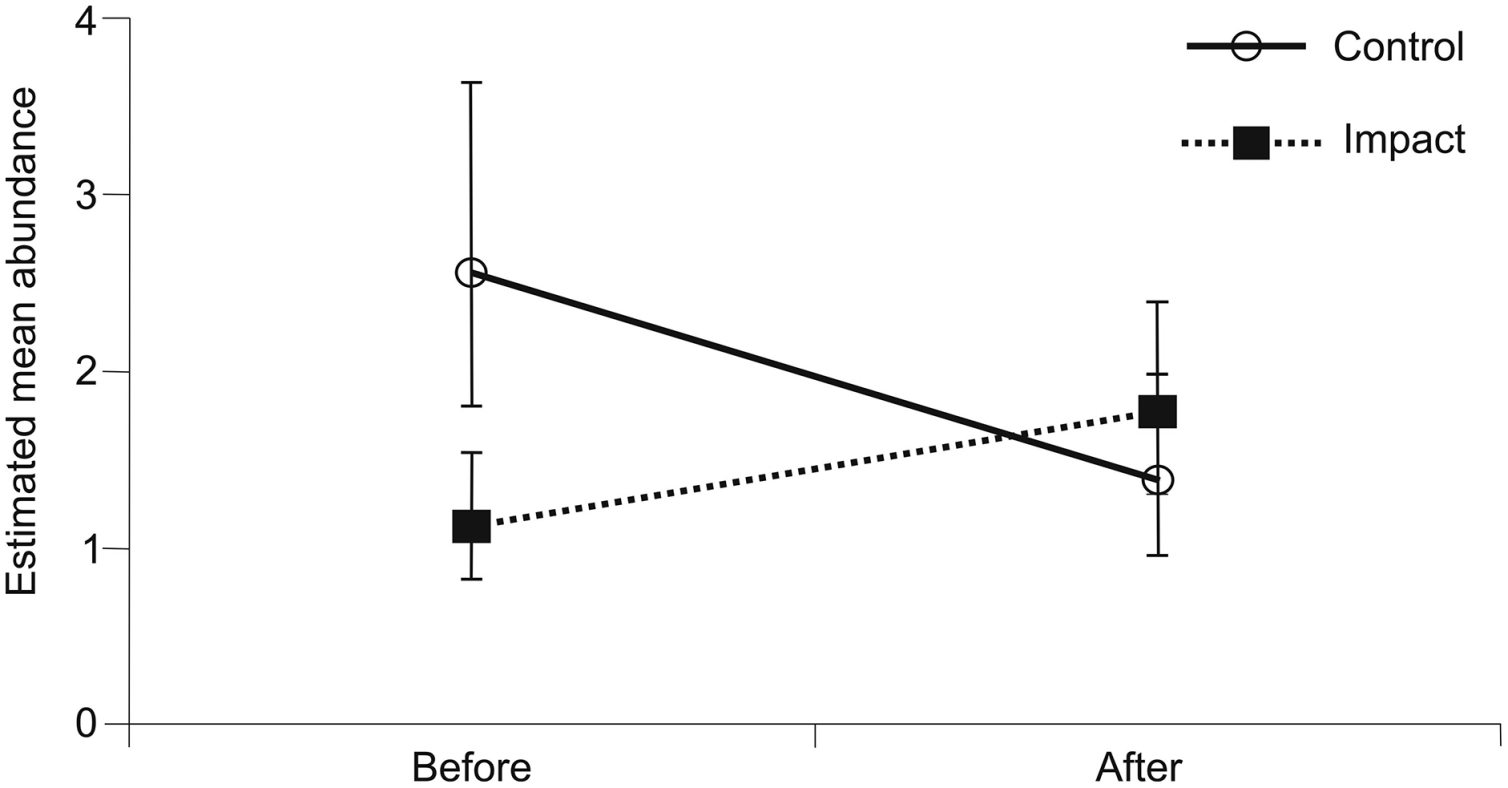
Estimated mean abundance of river fauna in the BACI design. The interaction is significant at *p* = 0.001.

After the dredging, a greater total number of species was found and many more individuals were caught than before the dredging (Table 2, Fig. 3). The GLMM results showed that the differences in abundance before and after the dredging were statistically significant (Table 3). More individuals were caught after the dredging in all taxa except Odonata (Table 2). More species were found in all groups except Heteroptera, in which the number of species remained unchanged. Hydrachnidia was the group with the greatest increase in the number of individuals and species. In the case of Odonata, the number of larvae caught before and after the dredging was similar, but a very pronounced decline in the number of adults was noted after the dredging (Table 2). There were also fewer species of adult Odonata after the dredging.

**Table 3:** GLMM model results for analysis of the abundance of all taxa.

Source	*F* statistics	df1	df2	Significance
Corrected model	4.611	3	826	0.003
Before-after	0.270	1	826	0.603
Control-impact	2.922	1	826	0.088
BA × CI	10.800	1	826	0.001

Some species that had been found in the river before the dredging were not caught after the dredging. At the same time, new species were found that had not been caught before the dredging (Table 4, Appendix 1). The most species that disappeared after the dredging were Coleoptera, while the most new species that had not been caught before the dredging belonged to the Hydrachnidia (Table 4).

**Table 4:** Numbers of species found in the river only before the dredging, only after the dredging, and both before and after the dredging.

Taxa	Only before the dredging	Only after the dredging	Both before and after the dredging
Mollusca	1	13 (1)	18 (1)
Hydrachnidia	6 (1)	40 (8)	23 (1)
Odonata larvae	0	3	7
Odonata adults	6	5 (1)	12 (1)
Heteroptera	3 (1)	3	15 (1)
Coleoptera	14	29 (1)	14
Trichoptera	2	14 (1)	10

**Note:**

Numbers in parentheses are the numbers of rare species within the category. For a detailed list of species, including rare species, see Appendix 1.

**Figure 4:**
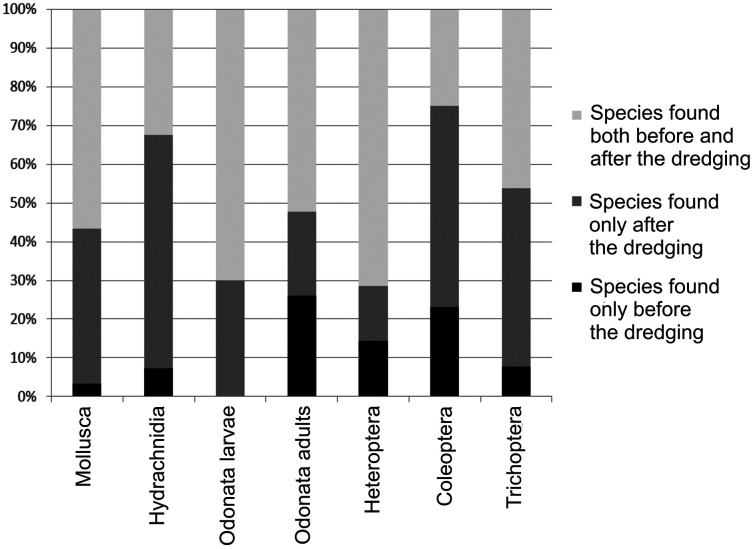
Percentage shares of the numbers of species found only before the dredging, only after the dredging, and both before and after the dredging in the total species richness within a given group.

The greatest change in species structure after the dredging was observed in beetles, as only 24.6% of the total number of Coleoptera species found in the river were caught both before and after the dredging (Fig. 4). Comparison of the changes in species composition before and after dredging in percentage terms shows that the greatest loss of species (26.1%) due to the intervention occurred in adult Odonata (Fig. 4).

**Figure 5:**
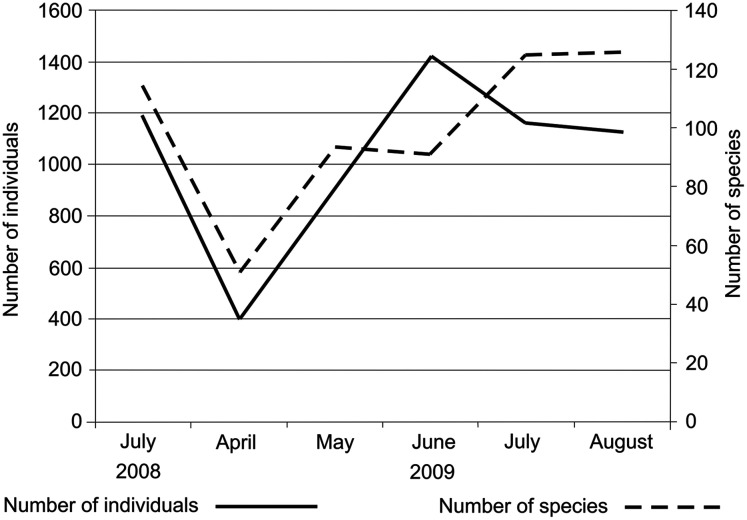
Changes in the numbers of all individuals and species caught in the Krąpiel before dredging (July 2008) and in successive months after dredging (April–August 2009).

After the dredging, the number of individuals in the river began to increase in April, reaching a maximum in June (Fig. 5). After that, the abundance of fauna remained fairly high in July and August. The differences in the number of individuals caught in each month after the intervention were statistically non-significant (Kruskal–Wallis test: H (4, *N* = 1,099) = 4.061550 *p* = 0.3977). The numbers of species caught after the dredging increased each month, reaching their maximum in August (Fig. 5).

**Figure 6:**
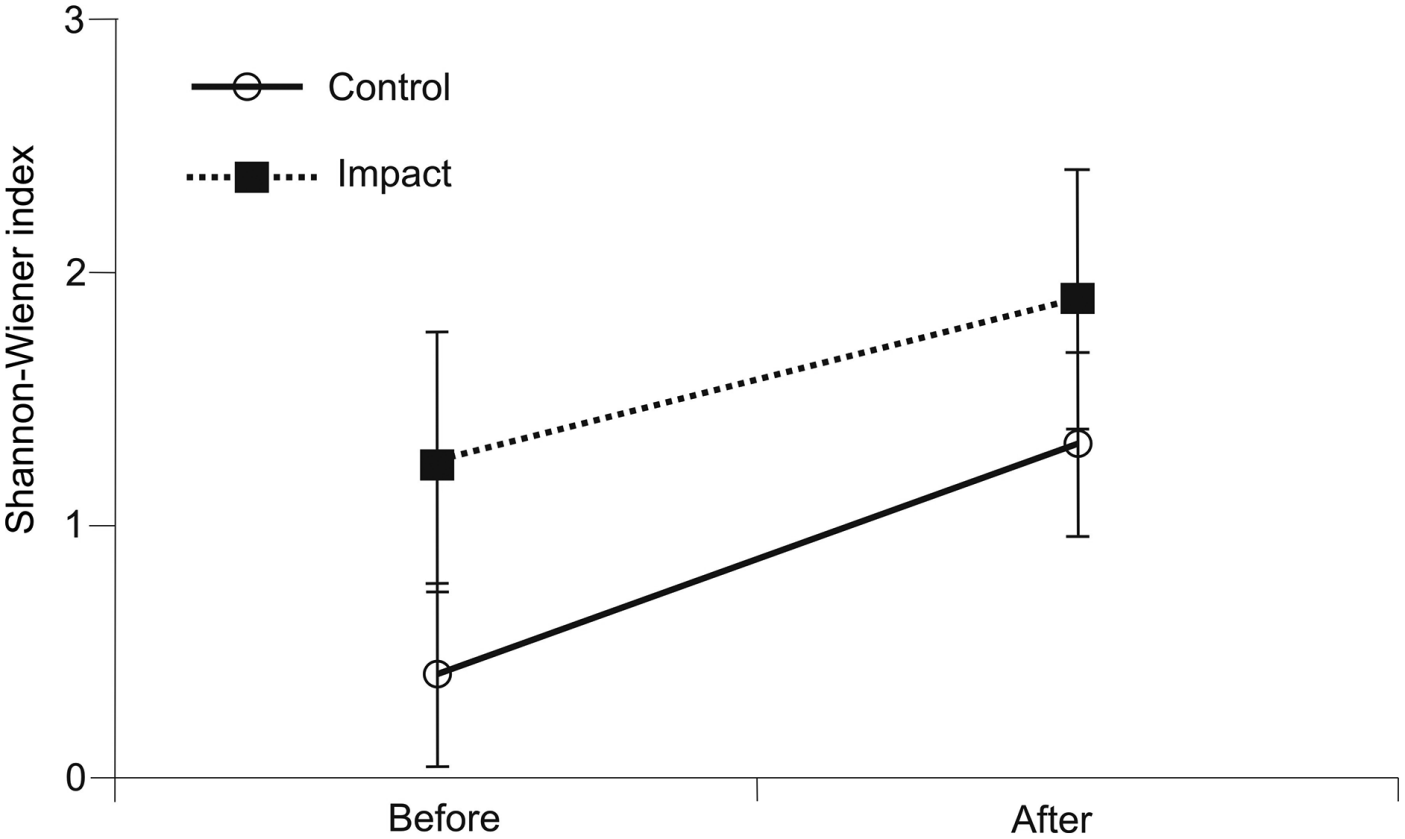
Shannon–Wiener index of river fauna diversity. The interaction was not significant (*p* > 0.05).

Species diversity was greater after the dredging than before it (Fig. 6), but the differences were not statistically significant (Table 5).

**Table 5:** Results of factorial ANOVA.

Source	Sum of squares	Df	Mean square	F-statistic	*p*
Intercept	9.428	1	31.697	130.938	0.000
Before-after	3.233	1	3.233	13.355	0.002
Control-impact	2.	1	2.665	11.008	0.003
BA × CI	0.097	1	0.097	0.403	0.533
Error	4.841	20	0.242		

**Figure 7:**
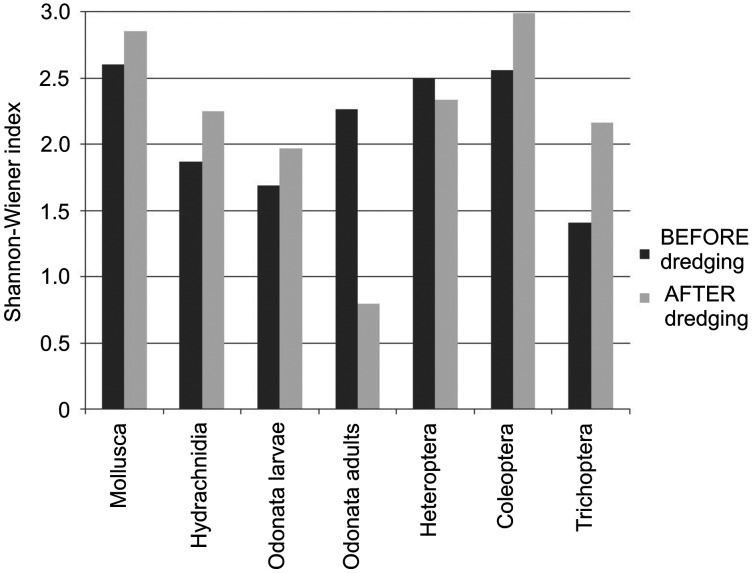
Shannon–Wiener index for individual taxa before and after dredging.

Of the seven analyzed invertebrate groups (including the division of Odonata into larvae and adults), species diversity was greater after the dredging in five groups (Mollusca, Hydrachnidia, Odonata larvae, Coleoptera and Trichoptera; Fig. 7). In two groups (Odonata adults and Heteroptera), species diversity decreased after the dredging, especially in the case of Odonata adults, as the Shannon-Wiener index decreased from 2.262 to 0.796 (Fig. 7).

## Discussion

### Changes in habitats

**Figure 8:**
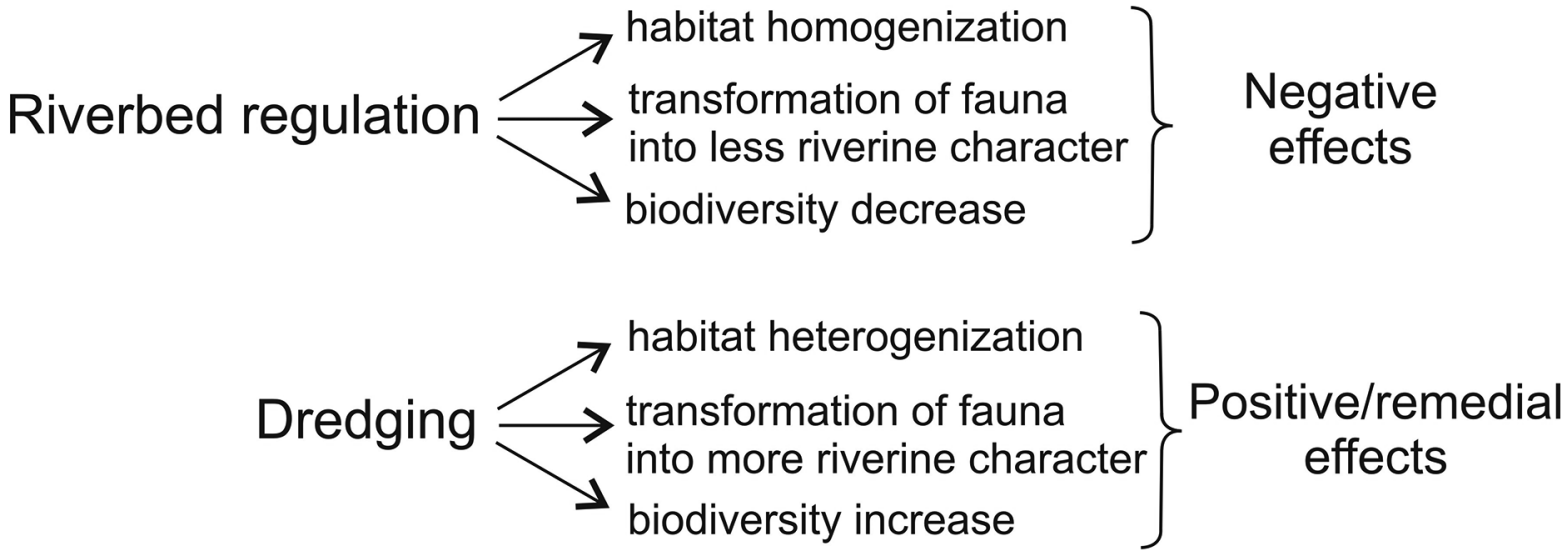
The effects of two stages of human impact on the investigated section of the River Krąpiel. River bed regulation–first stage of human impact, dredging–second stage of human impact.

**Figure 9:**
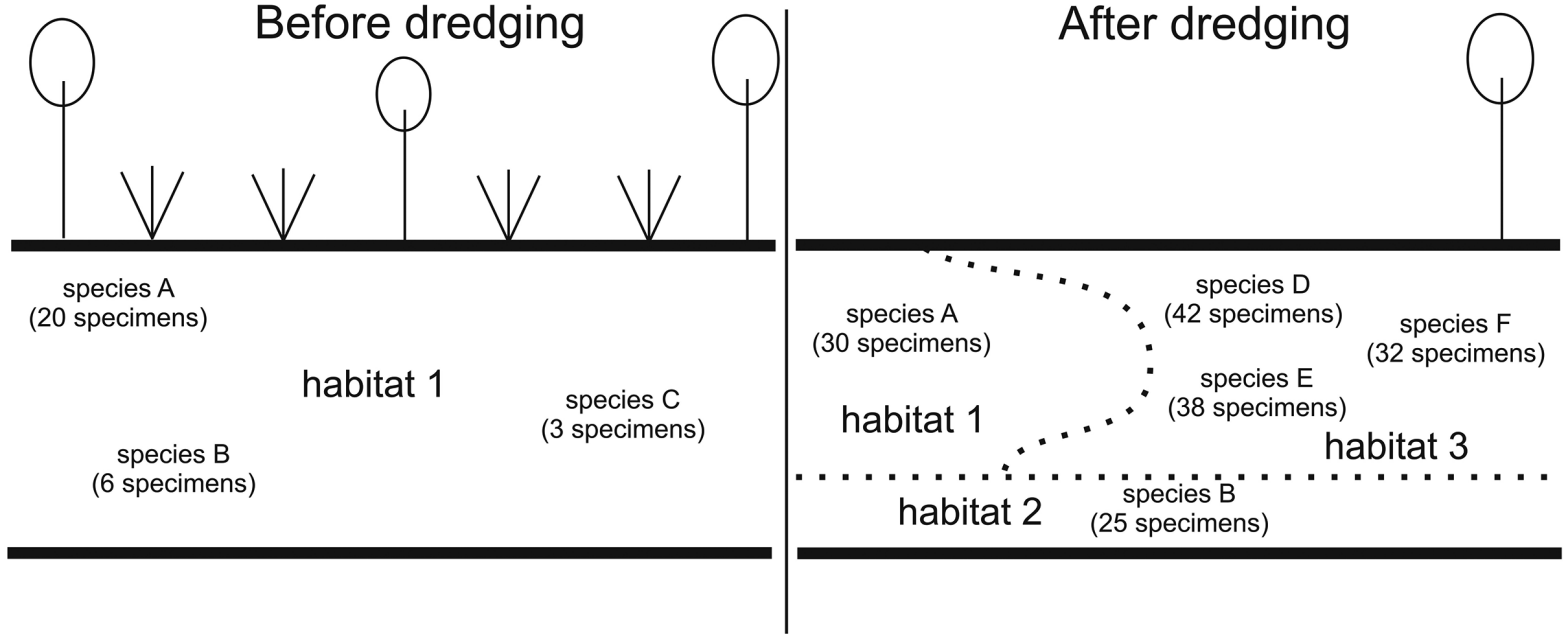
Diagram illustrating habitat and biocenotic changes in the investigated section of the River Krąpiel after dredging. After dredging there were more habitats (represented by numbers 1, 2, 3) than before dredging (represented by the number 1), more species were present (represented by letters A, B, D, E, F) than before dredging (A, B, C), and they were more numerous. Some species (C) were no longer present after dredging. Numbers and letters do not refer to any specific habitat or species, but only illustrate the general direction of the changes.

Human activity that physically alters the structure of river ecosystems, such as dredging, usually exerts negative effects as many niches are destroyed, thus reducing habitat diversity in the river (Allan & Flecker, 1993; Crook et al., 2015; Moog et al., 2018; Blake & Rhanor, 2020). However, instead of leading to habitat impoverishment, in some cases human impact on river ecosystems may have the opposite effect, as anthropogenic changes result in a new spatial structure of mesohabitats (Armitage & Pardo, 1995) and may lead to the formation of new habitats and niches (Feld, de Bello & Doledec, 2014; Płaska et al., 2016). In the case of the examined stretch of the Krąpiel, the dredging procedure was carried out on a previously regulated part of the river. The long-term effects of the prior river bed regulation were the homogenization of habitats, and the disappearance of some typical river habitats, *e.g.*, fast water current environments. As a consequence of these processes, the fauna of this stretch of the river before the dredging was not typical of rivers (Zawal et al., 2015; Buczyński et al., 2016; Dąbkowski et al., 2016; Płaska et al., 2016; Zawal et al., 2016a, 2016b). The changes resulting from the first stage of human impact, *i.e.*, river bed regulation, on the investigated section of the river can be considered unfavorable, as they had some negative effects on the river biotope (Fig. 8). As a result of dredging of the previously regulated stretch of the river, habitat diversity in the aquatic environment increased (Fig. 9), as a mosaic of habitats with varied macrophyte cover, varied sediments, and varied water current appeared. Thus, changes resulting from the second stage of human impact on the investigated section of the river, *i.e.*, dredging, can be considered favorable, as they had some positive effects on the river biotope (Fig. 8). Dredging of that stretch of the river can be said to have had a remedial effect, restoring its more natural character, similar to that of the unregulated, natural stretches of the river.

The terrestrial environment was negatively affected by the intervention. The removal of marginal vegetation led to a reduction in habitat diversity and the loss of ecological niches (Fig. 9). These changes adversely affected the biocenotic parameters of populations of taxa whose life cycle includes a terrestrial phase (see discussion below).

### Changes in invertebrate assemblages

Most studies on human impact altering the structure of river ecosystems indicate the negative effects of such activity on aquatic biocenoses (Kaenel & Uehlinger, 1999; Aldridge, 2000; Ohimain, Imoobe & Benka-Coker, 2005; Kändler & Seidler, 2013). However, some papers indicate no negative effects on the qualitative and quantitative structure of invertebrate fauna (Clarke & Tully, 2014), or even report an increase in the abundance, density, species richness and species diversity of aquatic invertebrates in stretches of a river subjected to transformations of human origin (Rader & Ward, 1988; Gorzel & Kornijów, 2007; Benitez-Mora & Camargo, 2014).

In the River Krąpiel, more individuals and species were caught after the dredging than before it. While some species disappeared due to the dredging, at the same time new species were found that had not been caught before the intervention, including rare species (Table 4, Appendix 1). Thus on balance the dredging had a positive impact on the species richness of that stretch of the river. The increase in habitat diversity after the dredging translated into an increase in the species diversity of most investigated groups. The restoration of habitats associated with the water current after the intervention caused a shift in the ecological nature of invertebrate communities towards more riverine fauna (Zawal et al., 2015; Buczyński et al., 2016; Dąbkowski et al., 2016; Płaska et al., 2016; Zawal et al., 2016a, 2016b). Hence the changes resulting from the second stage of anthropogenic impact, *i.e.*, dredging, can be considered beneficial for invertebrate communities, because, as in the case of habitat changes, they had positive effects on the river biocenosis, such as increasing the number of species and individuals caught and the species diversity (Figs. 8 and 9). In the case of deliberate river restoration, there is usually a large increase in the number of invertebrates caught and a slight increase in species diversity (Friberg et al., 2016). The same effects were achieved by dredging of the Krąpiel in the stretch of the river under study. Our results confirm the observations of some authors that dredging carried out in anthropogenically altered or anthropogenic ecosystems can cause beneficial changes in them and can even be considered a factor restoring the diversity of macroinvertebrate communities (McCabe, Hinton & Emmelt, 1998; Vermonden et al., 2011; Buczyńska & Buczyński, 2019).

Human activities associated with river maintenance are considerable disturbances, and their effects on the biological recovery of ecosystems can be extended in time (Petts, Armitag & Castella, 1993; Kenny & Rees, 1996). To accelerate recolonization processes and fauna regeneration after transformation works, some sections of the river should be left unchanged, and maintenance works should be carried out in autumn and winter (Gorzel & Kornijów, 2007). The dredging discussed in our study was carried out on a relatively short stretch of the river, and some locations within the managed section were left unchanged, ensuring the presence of refugia from which the dredged locations could be recolonized. Furthermore, the dredging was carried out in the winter, outside the growing season, so its impact on assemblages of fauna was smaller than if it had been carried out in spring or summer. For these reasons, the fauna in the examined section of the river regenerated very quickly after the intervention, within 4–6 months. In most of the analyzed groups, recolonization was mainly the result of drift (Zawal et al., 2015; Buczyński et al., 2016; Płaska et al., 2016; Zawal et al., 2016a). In the case of groups whose life cycle includes a terrestrial phase, another route of recolonization, in addition to drift, was the dispersal of adults, as discussed in Buczyński et al. (2016), Dąbkowski et al. (2016), Płaska et al. (2016) and Zawal et al. (2016a). In the case of Hydrachnidia, a significant factor influencing recolonization was the migration of water mites from neighboring fish ponds (Zawal et al., 2015).

The aerial stages of aquatic macroinvertebrates are highly sensitive to changes in terrestrial environments adjacent to aquatic ecosystems and wetlands (Anderson & Vondracek, 1999; Raebel et al., 2012). For some systematic groups, such as Odonata, the terrestrial environment is as important as the aquatic habitat, as it provides the conditions and resources required by adult dragonflies and damselflies (Corbet, 1999). Changes in the terrestrial environment may affect Odonata populations, as the species composition can vary depending on the presence of marginal vegetation (Balzan, 2012; da Silva Monteiro Júnior et al., 2013). Removal of riparian vegetation leads to a decrease in habitat diversity and may cause a reduction in the abundance of the adult Odonata population as well as homogenization of its species composition (Remsburg & Turner, 2009; de paiva Silva, Marco & Resende, 2010). In the case of the examined section of the Krąpiel, the removal of riparian vegetation led to drastic quantitative impoverishment of the adult Odonata community and loss of species. Of the invertebrate groups included in our study, Odonata proved particularly useful for assessing the impact of the intervention on invertebrate communities in this part of the river. Our results confirm literature data (Kutcher & Bried, 2014; Golfieri et al., 2016; Luke et al., 2017) indicating that Odonata are good indicators of human-induced habitat transformations, as their response to diverse environmental changes in both the aquatic habitat itself and the adjacent terrestrial environment is more comprehensive than that of other invertebrate groups.

Hydrachnidia, another group analyzed in our study, are generally hydrobionts, but their development cycle includes a terrestrial phase when larvae leave the aquatic environment (Davids et al., 2007). Most water mites parasitize Chironomidae (Smith & Oliver, 1986). As the terrestrial environment is an essential factor determining the Chironomidae population structure (Deletre & Morvan, 2000), changes in the terrestrial environment after the intervention (removal of riparian vegetation) may have affected populations of insect hosts of water mite larvae. This in turn may have influenced the number and species composition of water mite larvae returning to the river from their insect hosts. The Hydrachnidia community reacted to the investment to a lesser degree than the investigated groups of insects. One of the reasons for this less pronounced response could be the smaller role of the terrestrial phase in the life cycle of Hydrachnidia as compared to insects, and consequently a smaller impact of changes in the terrestrial environment on the Hydrachnidia community after dredging.

Of all groups investigated in this study, the smallest loss of species after the dredging was observed for Mollusca (only one species disappeared). Although mollusks can disperse through the aerial environment (Kappes & Haase, 2012), the role of the terrestrial phase in these invertebrates, compared to the other groups included in the study, should be considered negligible.

Regarding the percentage share of species that disappeared after the dredging in the total number of species found during the entire study period, we can see an upward trend from Mollusca (typical hydrobionts) to Hydrachnidia (which explore the aquatic environment as adults and the terrestrial environment as larvae) to insects (especially adult Odonata, which explore the terrestrial environment) (see Fig. 4). This pattern supports the two research hypotheses posed in this work: (1) the response of individual groups of investigated invertebrates to the intervention depends on the changes that have taken place in both the aquatic and terrestrial environment; (2) the greater the role of the terrestrial phase in the life cycle of a given group of invertebrates, the more they are affected by changes in the terrestrial environment following the intervention.

## Conclusions

1. The stretch of the River Krąpiel which is the subject of the present study had previously been transformed by human activity. Our results indicate that dredging carried out in a previously anthropogenically altered (regulated) section of the river can cause beneficial changes for the ecosystem. While some species disappeared due to the dredging, at the same time new species were found that had not been caught before the intervention, so on balance the dredging had a positive impact on invertebrate communities. Also, as a result of dredging, habitat diversity in the aquatic environment increased.
2. The response of individual groups of invertebrates to the intervention was strongly influenced by the role of the terrestrial phase in their life cycle: the greater the role of the terrestrial phase in the life cycle, the more the group was affected by changes in the terrestrial environment following the intervention.
3. Individual systematic groups showed diverse responses to the dredging. The dredging had the greatest impact on insects, and among these, on adult Odonata.

## Supplemental Information

10.7717/peerj.12224/supp-1 Supplemental Information 1 Raw data.

10.7717/peerj.12224/supp-2 Supplemental Information 2 Species found only before the dredging (A), only after the dredging (B) and both before and after the dredging (C) within each group of invertebrates. *–rare species.

## Additional Information and Declarations

### Field Study Permissions

The following information was supplied relating to field study approvals (*i.e*., approving body and any reference numbers): The research was made at the public/government areas where every person has a free access. To use those areas no permission was needed.

### Data Availability

The following information was supplied regarding data availability: The raw data is available in the Supplementary File.
