# Supplementary material for: Anthropogenic transformations of river ecosystems are not always bad for the environment: Multi-taxa analyses of changes in aquatic and terrestrial environments after dredging of a small lowland river
Source: PeerJ. 2021 Sep 29;9:e12224. doi: 10.7717/peerj.12224 (PMC8487244; doi:10.7717/peerj.12224)
Supplement: Supplemental Information 2 — *–rare species. [file peerj-09-12224-s002.docx]

**Mollusca**

A.

*Anodonta anatina*

B.

*Ancylus fluviatilis*

*Bithynia leachii*

*Cochlicopa lubricella*

*Gyraulus laevis**

*Physa fontinalis*

*Pisidium casertanum*

*Pisidium henslowanum*

*Pisidium ponderosum*

*Pisidium subtruncatum*

*Pisidium supinum*

*Planorbis carinatus*

*Potamopyrgus antipodarum*

*Radix auricularia*

C.

*Anisus vortex*

*Bithynia tentaculata*

*Galba truncatula*

*Gyraulus albus*

*Gyraulus crista*

*Lymnaea stagnalis*

*Pisidium amnicum*

*Planorbarius corneus*

*Planorbis planorbis*

*Radix ampla*

*Radix balthica*

*Segmentina nitida*

*Sphaerium corneum*

*Succinea elegans*

*Theodoxus fluviatilis*

*Unio crassus**

*Unio tumidus*

*Viviparus viviparus*

**Hydrachnidia**

A.

*Eylais discreta**

*Eylais mutila*

*Hydrochoreutes ungulatus*

*Piona rotundoides*

*Torrenticola dudichi*

*Unionicola crassipes*

B.

*Arrenurus albator*

*Arrenurus batillifer*

*Arrenurus bruzelli*

*Arrenurus conicus**

*Arrenurus crassicaudatus*

*Arrenurus falciger*

*Arrenurus globator*

*Arrenurus integrator*

*Arrenurus mediorotundatus*

*Arrenurus muelleri**

*Arrenurus sinuator*

*Arrenurus tubulator*

*Brachypoda montii**

*Eylais extendens*

*Hydrachna cruenta*

*Hydrachna globosa*

*Hydrochoreutes krameri*

*Hydrodroma pilosa**

*Hygrobates fluviatilis*

*Hygrobates foreli*

*Hygrobates trigonicus*

*Lebertia fimbriata*

*Lebertia longiseta*

*Lebertia natans*

*Lebertia oblonga*

*Lebertia porosa*

*Limnesia fulgida*

*Nautarachna crassa**

*Neumania callosa**

*Neumania limosa*

*Neumania papillosa**

*Piona alpicola*

*Piona carnea*

*Piona conglobata*

*Piona dispersa*

*Piona imminuta*

*Piona longipalpis*

*Piona paucipora*

*Pionopsis lutescens*

*Teutonia cometes**

C.

*Albia stationis**

*Atractides nodipalpis*

*Forelia variegator*

*Hydrodroma despiciens*

*Hydryphantes dispar*

*Hygrobates longipalpis*

*Hygrobates setosus*

*Lebertia inaequalis*

*Lebertia insignis*

*Lebertia rivulorum*

*Limnesia maculata*

*Limnesia undulata*

*Limnesia undulatoides*

*Mideopsis crassipes*

*Mideopsis orbicularis*

*Piona coccinea*

*Piona neumani*

*Piona pusilla*

*Piona variabilis*

*Sperchon clupeifer*

*Torrenticola amplexa*

*Unionicola aculeata*

*Unionicola gracilipalpis*

**Odonata larvae**

A.

-

B.

*Aeshna cyanea*

*Aeshna mixta*

*Somatochlora metallica*

C.

*Calopteryx splendens*

*Calopteryx virgo*

*Platycnemis pennipes*

*Ischnura elegans*

*Erythromma najas*

*Gomphus vulgatissimus*

*Sympetrum vulgatum*

**Odonata adults**

A.

*Coenagrion pulchellum*

*Crocothemis erythraea*

*Enallagma cyathigerum*

*Erythromma najas*

*Orthetrum cancellatum*

*Sympecma fusca*

B.

*Pyrrhosoma nymphula*

*Somatochlora flavomaculata*

*Sympetrum danae*

*Sympetrum depressiusculum**

*Sympetrum meridionale*

C.

*Calopteryx splendens*

*Calopteryx virgo*

*Coenagrion puella*

*Gomphus vulgatissimus*

*Ischnura elegans*

*Lestes sponsa*

*Platycnemis pennipes*

*Somatochlora metallica*

*Sympecma paedisca**

*Sympetrum flaveolum*

*Sympetrum sanguineum*

*Sympetrum vulgatum*

**Heteroptera**

A.

*Mesovelia furcata*

*Microvelia umbricola**

*Sigara distincta*

B.

*Callicorixa praeusta*

*Gerris odontogaster*

*Ranatra linearis*

C.

*Aphelocheirus aestivalis*

*Aquarius najas*

*Cymatia coleoptrata*

*Gerris argentatus*

*Gerris lacustris*

*Gerris rufoscutellatus*

*Hesperocorixa linnaei*

*Hesperocorixa sahlbergi*

*Hydrometra stagnorum**

*Ilyocoris cimicoides*

*Nepa cinerea*

*Notonecta glauca*

*Plea minutissima*

*Sigara falleni*

*Sigara striata*

**Coleoptera**

A.

*Cercyon bifenestratus*

*Colymbetes fuscus*

*Elmis aenea*

*Gyrrinus natator*

*Gyrrinus substriatus*

*Helochares lividus*

*Hydraena gracilis*

*Hydraticus seminiger*

*Hydroporus dorsalis*

*Hydroporus ferrugineus*

*Hydroporus planus*

*Laccobius hyalinus*

*Limnus volcmari*

*Ochthebius dilatatus*

B.

*Acilius canaliculatus*

*Acilius sulcatus*

*Agabus undulatus*

*Anacaena limbata*

*Anacaena lutescens*

*Coelambus imperssopunctataus*

*Cymbiodyta marginella*

*Dytiscus dimidiatus*

*Enochrus melanocephalus*

*Graptodytes pictus*

*Haliplus heydeni*

*Haliplus immaculatus**

*Hydraena palustris*

*Hydrochus carinatus*

*Hydroporus angustatus*

*Hydroporus palustris*

*Hygrotus inaequalis*

*Hyphydrus ovatus*

*Ilybius fenestratus*

*Ilybius fuliginosus*

*Ilybius guttiger*

*Ilybius subaeneus*

*Laccobius minutus*

*Laccobius striatulus*

*Noterus clavicornis*

*Noterus crassicornis*

*Ochthebius bicolon*

*Rhantus exoletus*

*Rhantus pulverosus*

C.

*Agabus bipustulatus*

*Haliplus flavicollis*

*Haliplus fluviatilis*

*Haliplus ruficollis*

*Helophorus dorsalis*

*Helophorus griseus*

*Helophorus minutus*

*Hydraena riparia*

*Hydrobius fuscipes*

*Ilybius ater*

*Ilybius obscurus*

*Laccophilus hyalinus*

*Laccophilus minutus*

*Oulimnius tuberculatus*

**Trichoptera**

A.

*Athripsodes cinereus*

*Lepidostoma hirtum*

B.

*Anabolia hybrida*

*Anabolia nervosa*

*Brachycentrus subnubilus*

*Ceraclea dissimilis*

*Goera pilosa*

*Halesus tesselatus*

*Hydropsyche incognita*

*Hydropsyche siltalai*

*Limnephilus flavicornis*

*Limnephilus rhombicus*

*Oecetis furva*

*Oecetis lacustris*

*Oecetis testacea**

*Polycentropus irroratus*

C.

*Halesus digitatus*

*Halesus radiatus*

*Hydropsyche angustipennis*

*Hydropsyche pellucidula*

*Limnephilus lunatus*

*Mystacides azurea*

*Neureclipsis bimaculata*

*Potamophylax latipennis*

*Potamophylax rotundipennis*

*Triaenodes bicolor*
